# Effectiveness of short term acute electroconvulsive therapy at three Brazilian sites: an observational cohort study

**DOI:** 10.1590/1516-3180.2023.0292.R1.03072024

**Published:** 2025-02-24

**Authors:** Leonardo Baldaçara, Mateus José Abdalla Diniz, Matheus Nycolas Barbosa de Andrade, Tiago Nunes de Araújo, Alfredo Minervino

**Affiliations:** I Professor, Department of Medicine, Universidade Federal do Tocantins (UFT), Palmas (TO), Brazil; Psychiatrist, Hospital Geral de Palmas, Palmas (TO), Brazil.; II Pax Instituto de Psiquiatria, Goiânia (GO), Brazil.; III Medical School. Universidade Federal da Paraíba (UFPB), João Pessoa (PB), Brazil.; IV Professor, Faculty of Medicine. Centro Universitário de João Pessoa (UNIPÊ), João Pessoa (PB), Brazil; Psychiatrist, Clínica Animus, João Pessoa (PB), Brazil.; V Professor, Department of Internal Medicine, Health Sciences Center, Universidade Federal da Paraíba (UFPB), João Pessoa (PB), Brazil; Psychiatrist, Clínica Animus, João Pessoa (PB), Brazil.

**Keywords:** Convulsive therapy, Electroconvulsive therapy, Mental Disorders, Psychiatric Somatic Therapies, Psychiatry, Treatment outcome, Psychiatric Illness, Treatment effectiveness, Response rate

## Abstract

**BACKGROUND:**

Electroconvulsive therapy (ECT) remains a target of prejudice, and finding places to administer it remains a challenge. The main reason for the recommendation of ECT is its effectiveness, especially when compared with other treatments for severe and refractory patients.

**OBJECTIVES:**

This study aimed to assess the response rates across a broader patient sample undergoing ECT in three distinct Brazilian states.

**DESIGN AND SETTING:**

This observational cohort study was conducted at the following three sites: Universidade Federal do Tocantins (by partnership with Hospital Geral de Palmas, Palmas-TO), Pax Instituto de Psiquiatria (Goiânia – GO), and Clínica Animus (Joao Pessoa – PB).

**METHODS:**

A total of 212 patients who received ECT at three different Brazilian services were assessed for improvement in symptoms in the first week after treatment and 30 and 60 days after treatment completion.

**RESULTS:**

Safety and efficacy of ECT was well established, as evidenced by the zero mortality rate among the study participants, with side effects observed in only 10.5% of cases. The immediate response rate was impressive at 95.8%, and the response rate after 30 and 60 days was 90.6% and 87.7%, respectively. The regression analysis highlighted session frequency as a key determinant of positive responses.

**CONCLUSION:**

The effectiveness of short term ECT (two months) is one of the greatest among psychiatric treatments. Future research should focus on predictive models for treatment responses to enable personalized approaches.

## INTRODUCTION

Since its establishment in 1938, electroconvulsive therapy (ECT) has been subjected to improvements with the objective of enhancing its therapeutic effectiveness while simultaneously reducing potential negative consequences.^1^ Currently, ECT is widely recognized and accepted as a safe and effective treatment option in the field of therapy. It is essential to follow precise procedural guidelines with great care and attention to detail. The responsibility of overseeing, implementing, and monitoring falls squarely within the domain of the medical professionals involved in its delivery.^2,3,4,5,6,7^

Some authors are still skeptical of its usefulness because of small impact size and high sample heterogeneity of the research. In addition, there are several factors unique to ECT that make it challenging to compare results. Most studies have shown efficacy; however, others have cast doubt. Regardless of diagnosis, a recent study found an instant response rate of 94.8% and a 30-day response rate of 84.5%.^6^ Besides, there is also a significant lack of public assistance for ECT, mainly affecting poor and severely ill patients.^8^

## OBJECTIVES

This study primarily aimed to examine the response rates of patients undergoing ECT in three distinct regions of Brazil (Tocantins, Goiás, and Paraíba). This investigation was motivated by the increasing challenges associated with accessing facilities that offer this procedure as well as the recognition of its effectiveness as a key factor for its indication. To achieve this objective, a larger sample size encompassing patients from three different healthcare services was used.

## METHODS

This observational cohort study investigated the effectiveness of ECT at three services in three separate states. Data were collected from the medical records of patients with various mental illnesses. All prospectively registered cases were assessed. The inclusion criteria were as follows: age ≥ 18 years, record of symptoms and medical records by scales, and record of other research variables. The exclusion criteria were patients with indications for ECT who did not undergo any sessions.

### Statistical analysis

The primary outcome measures consisted of three time points: immediate reaction (occurring within the initial 7 days), response at 30 days post-ECT, and response at 60 days post-ECT. The response was deemed an improvement in symptoms, as assessed by the Brief Psychiatric Rating Scale (BPRS), Altman Scale, and Hamilton Scale, with a minimum of 50% reduction in symptoms. The study considered several secondary characteristics including age, gender, diagnosis, number of sessions, side effects, and treatment discontinuation. Nominal variables were represented using numerical values and percentages. The use of continuous variables is a common practice for calculating measures of central tendency, such as the mean, and measures of dispersion, such as the standard deviation. The response rate was reported in both numerical and percentage forms, based on patients who demonstrated a minimum of 50% improvement in symptoms at each of the three time points.

A logistic regression analysis was conducted to ascertain the potential impact of secondary factors on the primary results. A significance level of 0.05 was established, which was adjusted to account for the number of variables included in each model.

### Ethical approval

This study protocol was approved by the Ethics Committee of the Universidade do Tocatins (CAAE: 68987823.0.0000.5519, Parecer: 6.085.547, 05/28/2023).

## RESULTS

The immediate response rate was 95.8%, and it was 90.6% and 87.7% after 30 and 60 days, respectively. Lower results were observed in Palmas and higher in João Pessoa. Detailed results are presented in Table 1.

**Table 1:** Total number of responses immediately, 30, and 60 days after electroconvulsive therapy at the three sites

Variable	Number n = 212	Immediate response n = 203 (95.8%)	30 days response n = 192 (90.6%)	60 days response n = 186 (87.7%)
Local				
Palmas	58 (27.4%)	54 (93.1%)	48 (82.8%)	45 (77.6%)
Goiânia	102 (48.1%)	99 (97.1%)	92 (90.2%)	89 (87.3%)
João Pessoa	52 (24.5%)	50 (96.2%)	52 (100.0%)	51 (98.1%)

When the response rate was calculated by diagnosis, the immediate response rate was 92.0% for schizophrenia, 97.3% for bipolar disorder, 91.7% for schizoaffective disorder, 100.0% for depressive disorder, 100.0% for induced psychosis, and 100.0% for organic mental disorder. After 30 days, the response rates by diagnosis was 86.7% for schizophrenia, 93.2% for bipolar disorder, 91.7% for schizoaffective disorder, 95.5% for depressive disorder, 33.3% for induced psychosis, and 100.0% for organic mental disorder. After 60 days, the response rates by diagnosis was 80.0% for schizophrenia, 91.9% for bipolar disorder, 91.7% for schizoaffective disorder, 95.5% for depressive disorder, 0% for induced psychosis, and 100.0% for organic mental disorder.

Demographic and clinical data are presented in Table 2. Logistic regression data to predict responses are presented in Table 3.

**Table 2:** Demographic and clinical data of all participants

Variable	Values (n = 212)
**Gender**	
Male (n, %)	113 (53.3%)
Female (n, %)	99 (46.7%)
**Age** (Mean ± SD)	38.4 ± 15.5
**Diagnosis**	
Schizophrenia (n, %)	75 (35.4%)
Bipolar disorder (n, %)	74 (34.9%)
Schizoaffective disorder (n, %)	12 (5.7%)
Depressive disorder (n, %)	44 (20.8%)
Puerperal psychosis (n, %)	2 (0.9%)
Induced psychosis (n, %)	3 (1.4%)
Organic mental disorder (n, %)	2 (0.9%)
**Substance use disorder**	
No (n, %)	190 (89.6%)
Yes (n, %)	22 (10.4%)
**Personality disorder**	
No (n, %)	201 (94.8%)
Yes (n, %)	11 (5.2%)
Indication	
Resistant (n, %)	130 (61.3%)
Pregnancy (n, %)	5 (2.4%)
Suicide behavior (n, %)	14 (6.6%)
Severity (n, %)	59 (27.8%)
Catatonia (n, %)	2 (0.9%)
Severe depression (n, %)	2 (0.9%)
**Adverse effects**	
No (n, %)	205 (96.7%)
Yes (n, %)	6 (2.8%)
Backpain (n, %)	2 (0.9%)
Headache (n, %)	1 (0.5%)
Muscle pain (n, %)	3 (1.4%)
Agitation after crisis (n, %)	1 (0.5%)
**Dropout**	
No (n, %)	206 (97.2%)
Yes (n, %)	6 (2.8%)
**Mean number of sessions** (Mean ± SD)	8.3 ± 5.3

**Table 3:** Results of logistic regression analysis to predict responses

Variable	Immediate	After 30 days	After 60 days
**Demographic**			
Age	Exp(B) = 1.018, P = 0.487	Exp(B) = 1.024, P = 0.192	Exp(B) = 1.042, P = 0.022
Gender	Exp(B) = 3.143, P = 0.160	Exp(B) = 2.145, P = 1.135	Exp(B) = 2.781, P = 0.029
**Clinical**			
Diagnosis	Exp(B) = 2.537, P = 0.061	Exp(B) = 0.934, P = 0.654	Exp(B) = 0.961, P = 0.770
Substance use disorder	Exp(B) = 0.912, P = 0.934	Exp(B) = 0.248, P = 0.014	Exp(B) = 0.283, P = 0.016
Personality disorder	Exp(B) = 0.234, P = 0.226	Exp(B) = 1.050, P = 0.966	Exp(B) = 1.494, P = 0.715
Indication	Exp(B) = 0.916, P = 0.719	Exp(B) = 1.366, P = 0.147	Exp(B) = 1.257, P = 0.193
**Technical**			
Number of sessions	Exp(B) = 1.085, P = 0.288	Exp(B) = 1.364, P < 0.001	Exp(B) = 1.235, P < 0.001
**Complications**			
Adverse effects	-	-	Exp(B) = 0.884, P = 0.724
Dropout	Exp(B) = 0.031, P < 0.001	Exp(B) = 0.016, P < 0.001	Exp(B) = 0.062, P = 0.002

Abandonment was a risk factor for immediate response. For response 30 days after finishing ECT, Substance use disorder (SUD) and abandonment were risk factors for a good outcome, and the number of sessions was a protective factor. For response 60 days after completing ECT, age, sex, and number of sessions were protective factors, and TUS and abandonment were risk factors for a favorable outcome.

A new logistic model with only significant variables for response 30 days after completing ECT was performed, and the number of sessions was a protective factor [Exp(B) 1.293, P = 0.006)] and abandonment a risk factor in this model [Exp(B) 0.036, P = 0.005)]. A new logistic model was used to assess the response 60 days after the completion of ECT. The number of sessions [Exp(B) = 1.205, P = 0.003] was the only protective factor in this model.

## DISCUSSION

ECT elicit response rates between 10% and 75% for acute schizophrenia and between 10% and 90% for chronic schizophrenia, depending on the specific parameters employed. In addition to symptom manifestation, ECT has shown efficacy in diminishing the need for physical restraint and facilitating prompt reassurance. Additionally, it had the ability to enhance the rate of discharge in a range of 10-22% based on the instances examined.^9,10,11,12,13^ Early ECT may reduce length of hospital stay without increasing total hospitalization costs or fatal adverse events in patients with major depressive disorder.^14^ The response rate in individuals with depression varied between 44.4% and 90%, with ECT demonstrating a more favorable response compared to antidepressant medications.^9,15,16,17,18,19,20,21,22,23^ For mania, the response rate varied between 80% and 92.3%.^15,24^ In comparison to our sample, Brazil exhibited greater response rates, particularly for conditions such as schizophrenia, schizoaffective disorder, bipolar disorder, and depression.

We found that a greater number of ECT sessions was associated with better results. Recent research has demonstrated increased efficacy with increasing doses (up to 12 times); however, clinically, increasing doses is limited by a commensurate increase in cognitive side-effects.^12,25^ In another investigation, patients with major depressive disorder (MDD) exhibited distinct and clinically relevant response trajectories to ECT. Patients with MDD with more severe depression at baseline were associated with a rapid response trajectory. In contrast, patients with MDD with severe symptoms and older age had a lower response trajectory. Future investigations should prioritize the identification of factors that may predict favorable outcomes in the context of personalized therapeutic interventions.^26^

In addition to our dataset, unsatisfactory outcomes have been frequently observed in the literature. A modest body of research supports the utilization of this intervention, particularly when employed in conjunction with antipsychotic medications for individuals diagnosed with schizophrenia who exhibit suboptimal response to pharmacotherapy in isolation.^27^ Moderate-quality evidence indicates that relative to standard care, ECT has a positive effect on medium-term clinical response for people with treatment-resistant schizophrenia. However, there are no clear and convincing advantages or disadvantages of adding ECT to standard care for other outcomes. The available evidence is too weak to indicate whether adding ECT to standard care is superior or inferior to adding sham ECT or other antipsychotics, and there is insufficient evidence to support or refute the use of ECT alone. Better-quality evidence is required before firm conclusions can be made.^28^

The varied outcomes observed in ECT in the literature can be attributed to variations in the protocols used. Among other factors, various services may have distinct indications, response criteria, device settings, and diverse methods of administering anesthesia. This observation indicates that many investigations on ECT are influenced by heterogeneity.

Regarding side effects, our rate was 10.5%, which was not severe. In the literature, the most frequent side effects observed were headache after the crisis in 45% and nausea in 1-23% of patients.^15^ Total side effects was reported in 14% patients with schizophrenia.^13^ As for other side effects, cardiovascular, pulmonary, and cerebrovascular events are reported; these may be minimized by screening for risk factors and physiologic monitoring. Although most cognitive adverse effects of ECT are short-lasting, troublesome retrograde amnesia and other cognitive symptoms may rarely persist.^7,23,29^ Moreover, sometimes, patients show more subjective than objective cognitive adverse effects of ECT.^30^ As for fear of mortality, our sample had no fatal cases. In remote literature, mortality has been reported in 0.1 to 0.3% of samples from over 500 patients.^9^

This study has two inherent limitations that require further consideration and resolution. Initially, the patients’ assessments were constrained to brief post-procedural follow-ups with a duration not exceeding 2 months. This limitation is a direct result of the need to effectively manage patients referred from many healthcare facilities to the three treatment programs in question. Furthermore, to assess the global response rate to ECT across various settings, the potential impact of additional factors such as the specific anesthetic employed or the drug supplied to the patients was not considered. Additional investigations are necessary to address these limitations, including conducting longitudinal follow-up assessments many months after the administration of electroconvulsive treatment, and including other types of short- and long-term medications. However, a significant advantage of this study is its robust sample size and incorporation of a multicenter sample, thus demonstrating its ability to replicate the findings across several locations.

## CONCLUSION

ECT demonstrated both safety and efficacy. The observed mortality rate in the tested group was zero; however, the incidence of adverse effects was 10.5%. The initial response rate was 95.85%, which decreased to 90.6% after 30 days and further decreased to 87.7% after 60 days. The regression analysis revealed that the increased number of sessions had the strongest association with response variable. Although further studies with extended follow-up are required, the existing literature suggests that a minimum of 12 sessions is recommended to obtain improved response rates and longer-lasting effects. ECT did not yield any discernible benefits in the context of psychosis.

## References

[1] Abrams R (2002). Stimulus titration and ECT dosing. J ECT.

[2] Baldaçara LR, Fidelis FAP, Fidalgo TM (2022). Matriz de competências em psiquiatria. Debates em Psiquiatria.

[3] Comissão Nacional de Residência Médica. (2021). Resolução CNRM n. 18, de 6 de julho de 2021. Aprova a matriz de competências dos programas de Residência Médica em Psiquiatria no Brasil.. Diário Oficial da União.

[4] Conselho Federal de Medicina. (2002). Resolução CFM n. 1.640. Dispõe sobre a eletroconvulsoterapia e dá outras providências.. Diário Oficial da União..

[5] Conselho Federal de Medicina. Resolução CFM n. 2.057. (2013). Consolida as diversas resoluções da área da Psiquiatria e reitera os princípios universais de proteção ao ser humano, à defesa do ato médico privativo de psiquiatras e aos critérios mínimos de segurança para os estabelecimentos hospitalares ou de assistência psiquiátrica de quaisquer naturezas, definindo também o modelo de anamnese e roteiro pericial em psiquiatria.. Diário Oficial da União..

[6] Ferroli B, Baldaçara L (2023). History of the implementation of electroconvulsive therapy at the General Public Hospital of Palmas. RSD.

[7] Stella F, Radanovic M, Gallucci J, Forlenza OV (2023). Electroconvulsive therapy for treating patients with agitation and related behavioral disorders due to dementia: a systematic review.. Dement Neuropsychol.

[8] Ribeiro RB, Melzer-Ribeiro DL, Rigonatti SP, Cordeiro Q (2012). Electroconvulsive therapy in Brazil after the “psychiatric reform”: a public health problem — example from a university service. J ECT.

[9] Turek IS, Hanlon TE (1977). The effectiveness and safety of electroconvulsive therapy (ECT). J Nerv Ment Dis.

[10] Petrides G, Malur C, Braga RJ (2015). Electroconvulsive therapy augmentation in clozapine-resistant schizophrenia: a prospective, randomized study.. Am J Psychiatry.

[11] Phutane VH, Thirthalli J, Muralidharan K (2013). Double-blind randomized controlled study showing symptomatic and cognitive superiority of bifrontal over bitemporal electrode placement during electroconvulsive therapy for schizophrenia. Brain Stimul.

[12] Ali SA, Mathur N, Malhotra AK, Braga RJ (2019). Electroconvulsive therapy and schizophrenia: a systematic review. Mol Neuropsychiatry.

[13] Lally J, Tully J, Robertson D (2016). Augmentation of clozapine with electroconvulsive therapy in treatment resistant schizophrenia: A systematic review and meta-analysis. Schizophr Res.

[14] Yamazaki R, Ohbe H, Matsuda Y (2021). Early electroconvulsive therapy in patients with major depressive disorder: a propensity score-matched analysis using a nationwide inpatient database in Japan. J ECT.

[15] Payne NA, Prudic J (2009). Electroconvulsive therapy: Part I. A perspective on the evolution and current practice of ECT. J Psychiatr Pract.

[16] Frey R, Schreinzer D, Heiden A, Kasper S (2001). Use of electroconvulsive therapy in psychiatry. Nervenarzt.

[17] van Duist M, Spaans HP, Verwijk E, Kok RM (2020). ECT non-remitters: prognosis and treatment after 12 unilateral electroconvulsive therapy sessions for major depression. J Affect Disord.

[18] Yildiz A, Mantar A, Simsek S (2010). Combination of pharmacotherapy with electroconvulsive therapy in prevention of depressive relapse: a pilot controlled trial. J ECT.

[19] Spaans HP, Verwijk E, Comijs HC (2013). Efficacy and cognitive side effects after brief pulse and ultrabrief pulse right unilateral electroconvulsive therapy for major depression: a randomized, double-blind, controlled study. J Clin Psychiatry.

[20] Pluijms EM, Kamperman AM, Hoogendijk WJG, van den Broek WW, Birkenhager TK (2022). Influence of adjuvant nortriptyline on the efficacy of electroconvulsive therapy: a randomized controlled trial and 1-year follow-up. Acta Psychiatr Scand.

[21] UK ECT Review Group (2003). Efficacy and safety of electroconvulsive therapy in depressive disorders: a systematic review and meta-analysis. Lancet.

[22] Carvalho I, Da Silva YH (2023). Eletroconvulsoterapia para depressão unipolar não responsiva: uma revisão bibliográfica. Braz J Health Rev.

[23] Landry M, Moreno A, Patry S, Potvin S, Lemasson M (2021). Current practices of electroconvulsive therapy in mental disorders: a systematic review and meta-analysis of short and long-term cognitive effects. J ECT.

[24] Mohan TS, Tharyan P, Alexander J, Raveendran NS (2009). Effects of stimulus intensity on the efficacy and safety of twice-weekly, bilateral electroconvulsive therapy (ECT) combined with antipsychotics in acute mania: a randomised controlled trial. Bipolar Disord.

[25] Loo CK, Schweitzer I, Pratt C (2006). Recent advances in optimizing electroconvulsive therapy. Aust N Z J Psychiatry.

[26] Su L, Zhang Y, Jia Y (2023). Predictors of electroconvulsive therapy outcome in major depressive disorder. Int J Neuropsychopharmacol.

[27] Tharyan P, Adams CE (2002). Electroconvulsive therapy for schizophrenia.. Cochrane Database Syst Rev.

[28] Sinclair DJ, Zhao S, Qi F (2019). Electroconvulsive therapy for treatment-resistant schizophrenia. Cochrane Database Syst Rev.

[29] Andrade C, Arumugham SS, Thirthalli J (2016). Adverse effects of electroconvulsive therapy. Psychiatr Clin North Am.

[30] Hammershøj LG, Petersen JZ, Jensen HM, Jørgensen MB, Miskowiak KW (2022). Cognitive adverse effects of electroconvulsive therapy: a discrepancy between subjective and objective measures?. J ECT.

